# In Vitro Evaluation of the Compatibility of Meropenem and Tanreqing Under Parallel Infusion Conditions

**DOI:** 10.3390/pharmaceutics18050596

**Published:** 2026-05-13

**Authors:** Xiaokai Ren, Xiao Li, Zhanjun Dong

**Affiliations:** 1 School of Pharmacy, Hebei Medical University, Shijiazhuang 050017, China; rxk15511053145@163.com; 2 Graduate School, Hebei Medical University, Shijiazhuang 050017, China; 3 Hebei Key Laboratory of Clinical Pharmacy, Department of Pharmacy, Hebei General Hospital, Shijiazhuang 050051, China; 15132106723@163.com

**Keywords:** meropenem, Tanreqing Injection, severe pneumonia, chlorogenic acid, compatibility

## Abstract

**Objective**: This study evaluated the in vitro physicochemical compatibility of meropenem and Tanreqing Injection under simulated parallel infusion conditions, providing experimental evidence to address existing gaps in compatibility data under clinically relevant co-infusion conditions. **Methods**: To simulate clinical dosages, meropenem and Tanreqing Injection were prepared individually and in combination in 100 mL and 250 mL of 0.9% sodium chloride injection (NS). Changes in appearance, pH, osmolality, insoluble particles, drug content, and related impurities were investigated over a 0–24 h period to assess the compatibility of each preparation by using a fully validated liquid chromatography method. **Result**: Meropenem alone exhibited a slow degradation trend over 24 h at room temperature. For Tanreqing Injection alone, the chlorogenic acid content decreased to 80.0% in 100 mL of NS and 85.2% in 250 mL of NS within 24 h, indicating improved stability at higher dilution volumes. When meropenem was combined with Tanreqing Injection, the chlorogenic acid content exhibited an immediate and significant decrease upon mixing. By 24 h, the reduction reached 71.9% in the 100 mL NS combination group and 44.0% in the 250 mL NS combination group. Concurrently, levels of meropenem Impurity A increased significantly in both combination groups, with more pronounced changes observed in the 100 mL NS group (*p* < 0.05). **Conclusions**: The parallel co-infusion of meropenem and Tanreqing Injection results in immediate chemical incompatibility and significant active constituent degradation. To ensure therapeutic efficacy and patient safety, simultaneous admixture of these two agents is strictly contraindicated in clinical practice. When sequential administration is necessary, a larger diluent volume (e.g., 250 mL NS) is preferred for Tanreqing Injection, and infusion lines must be thoroughly flushed between administrations to prevent residual interactions.

## 1. Introduction

Severe pneumonia (SP) is a leading critical illness in the intensive care unit (ICU), characterized by rapid progression and a high risk of multiple organ dysfunction. The mortality rate for SP complicated by septic shock ranges from 30 to 50% [1]. Prompt initiation of anti-infective therapy is the cornerstone of halting progression and improving patient outcome [2]. Meropenem, a second-generation carbapenem antibiotic, exhibits broad-spectrum activity against Gram-positive bacteria (including methicillin-resistant Staphylococcus aureus), Gram-negative bacteria (such as Pseudomonas aeruginosa and Acinetobacter baumannii), and anaerobes due to its unique β-lactam structure. Consequently, it is recommended as a first-line anti-infective regimen for SP in clinical practice [3,4,5,6]. In ICU practice, polypharmacy and the use of shared intravenous access systems (e.g., Y-site connectors and extension tubing) are routine. Although drugs are often prescribed separately, unintentional mixing within infusion lines prior to systemic administration is frequently unavoidable, creating potential risks of physicochemical incompatibility that may compromise drug stability and patient safety [7].

In recent years, the clinical integration of standardized, multicomponent bioactive formulations as adjunctive treatments has gained significant traction for modulating dysregulated immune responses in severe infections [8]. Tanreqing Injection is a modern Chinese patent medicine containing baicalin, chlorogenic acid, ursodeoxycholic acid, and bilirubin [9,10]. Contemporary pharmacological evidence indicates that these active constituents exert multi-target anti-inflammatory effects, thereby accelerating the resolution of pulmonary inflammation and improving arterial oxygenation when combined with meropenem [11,12,13,14]. Multiple clinical guidelines and expert consensuses advocate for the combination of meropenem and Tanreqing Injection for SP. Compared with monotherapy, this combination regimen can alleviate symptoms such as cough, expectoration, and wheezing; improve arterial blood gas indices (PaO_2_), hematological parameters (white blood cell count, C-reactive protein), and pulmonary function; accelerate the resolution of pulmonary inflammation; shorten hospital stays and the duration of antibiotic administration; and reduce antibiotic-related adverse reactions while enhancing overall clinical efficacy [15,16]. Despite its documented clinical benefits, systematic data regarding the compatibility and safety of mixing these two agents are insufficient. The stability of active ingredients, the formation of impurities, and the potential risks of adverse reactions following mixing remain unclear.

Intravenous drug incompatibility during simultaneous infusion is a widely recognized global challenge in critical care pharmacotherapy [17]. The unique β-lactam structure of meropenem renders it highly susceptible to degradation, ring-opening, and polymerization, particularly in the presence of fluctuating pH or reactive organic acids [18,19]. The physical mixing of sensitive carbapenems with multicomponent polyphenolic formulations in extension tubing introduces unpredictable physicochemical interactions, such as transient pH shifts, subvisible particulate formation, and the rapid degradation of active pharmaceutical ingredients. Such incompatibilities not only compromise the therapeutic efficacy of the antimicrobial regimen but also pose severe patient safety risks, including microvascular embolism, phlebitis, and catheter occlusion. International surveys of ICU nurses highlight that the scarcity of compatibility data for commonly used drugs is a significant challenge to standardized medication administration in critical care [20]. This has led to increasing global concern regarding drug compatibility in critically ill patients [21]. While the clinical efficacy of combined meropenem and Tanreqing Injection for SP is well-documented, no studies have evaluated their compatibility during parallel infusion.

To address this critical knowledge gap, this study systematically investigates the physicochemical compatibility of meropenem and TRQ under simulated, clinically relevant concurrent infusion conditions. Using common clinical diluents and concentrations, changes in visual appearance, pH, osmolarity, subvisible particulates (in accordance with pharmacopoeial standards), and the stability of specific marker components were dynamically monitored over 24 h. By quantitatively evaluating potential active component degradation and impurity formation (such as meropenem polymers), this research provides essential, experimentally validated evidence to optimize sequential or concurrent infusion protocols, mitigate compatibility-associated risks, and enhance medication safety for critically ill patients subjected to complex polypharmacy regimens.

## 2. Materials and Methods

### 2.1. Materials

The following analytical instruments were employed for the physicochemical characterization and quantitative analysis of the drug samples: H-CLASS ultra performance liquid chromatography (UPLC) (Waters Corporation, MA, USA); pHS-3C precision pH meter (Mettler-Toledo Instruments (Shanghai) Co., Ltd., Shanghai, China); MS 105 electronic analytical balance (Mettler-Toledo International Inc., Greifensee, Switzerland; readability: 0.01 mg); GWF-8JD particle counter (Tianjin Tianhe Analytical Instrument Co., Ltd.,Tianjin, China); and SMC 30C-1 osmometer (Tianjin Tianhe Analytical Instrument Co., Ltd., Tianjin, China).

### 2.2. Reagents and Drugs

The drugs, reagents, and materials utilized in this study, including their specifications and batch numbers, are as follows: Tanreqing Injection (Shanghai Kaibao Pharmaceutical Co., Ltd., Shanghai, China; specification: 10 mL; batch number: 2408306); meropenem for injection (PKU Pharma Co., Ltd., Shanghai, China; specification: 0.5 g; batch number: 240111); 0.9% sodium chloride injection (Shijiazhuang No. 4 Pharmaceutical Co., Ltd., Shijiazhuang, China; specification: 100 mL; batch number: 241043701); 0.9% sodium chloride injection (Shijiazhuang No. 4 Pharmaceutical Co., Ltd., Shijiazhuang, China; specification: 250 mL; batch number: 2412293704); 0.22 μm hydrophilic filter membrane (Beijing Solarbio Science & Technology Co., Ltd., Beijing, China; specification: 25 mm; batch number: 20240516); potassium dihydrogen phosphate (Tianjin Damai Chemical Reagent Factory, Tianjin, China; batch number: 20220601); phosphoric acid (Tianjin Yongda Reagent Co., Ltd., Tianjin, China; specification: 500 mL; batch number: 20231017); acetonitrile (chromatographic grade); and purified water.

### 2.3. Methods

#### 2.3.1. Chromatographic Conditions

Chromatographic separation was performed on a WondaSil C18 Superb column (5 μm, 4.6 mm × 150 mm; GL Sciences Trading Co., Ltd., Shanghai, China). The mobile phase consisted of acetonitrile (A) and 0.01% aqueous potassium dihydrogen phosphate (adjusted to pH 2.5 with phosphoric acid) (B). The gradient elution program was as follows: 0–30 min, 8% A; 30–40 min, linear gradient from 8% to 20% A; 40–55 min, linear gradient from 20% to 30% A; and 55–60 min, linear gradient from 30% to 2% A. The flow rate was maintained at 1.0 mL·min^−1^, the column temperature was set at 30 °C, and the detection wavelength was 318 nm. The injection volume was 10 μL.

#### 2.3.2. Physicochemical Evaluation

Visual inspection of the solutions was performed in accordance with the general chapters 0901 (Color of Solution), 0902 (Test for Clarity of Solution), and 0904 (Test for Visible Particulates) of the Chinese Pharmacopoeia (2025 Edition) [22]. Observations were conducted in a clean laboratory environment under controlled conditions (illuminance: 1500 lx; temperature: 25 ± 2 °C). Colorless transparent glass bottles were used for all evaluations to ensure consistent visual assessment. The solutions exhibited the characteristic brownish-yellow color of Tanreqing Injection, with no abnormal changes observed during the study.

pH was measured using a pHS-3C precision pH meter (Mettler-Toledo Instruments, Shanghai, China). The instrument was calibrated with standard buffer solutions (pH 4.00, 6.86, and 9.18) before measurement. All measurements were performed at 25 ± 1 °C, and each sample was analyzed in triplicate. Osmolality was determined using an SMC 30C-1 freezing-point osmometer (Tianjin Tianhe Analytical Instrument Co., Ltd., Tianjin, China). The instrument was calibrated with standard solutions prior to use. Samples were equilibrated at room temperature for 10 min before measurement, and each sample was measured in triplicate.

Sub-visible particulate analysis was performed using the light obscuration method in accordance with General Chapter 0903 of the Chinese Pharmacopoeia (2025 Edition) [22], employing a GWF-8JD particle counter (Tianjin Tianhe Analytical Instrument Co., Ltd., China). Prior to measurement, samples were allowed to stand undisturbed for 5 min to eliminate air bubbles. For each sample, the initial measurement was discarded to ensure system equilibration, and the final result was calculated as the mean of three subsequent measurements. Acceptance criteria for compatibility were defined as ≤25 particles/mL for particles ≥10 μm and ≤3 particles/mL for particles ≥25 μm.

### 2.4. Preparation of Solutions

#### 2.4.1. Reference Stock Solutions

Appropriate amounts of baicalin, chlorogenic acid, and meropenem impurity A reference standards were accurately weighed and transferred into separate 10 mL volumetric flasks. The standards were dissolved and diluted to volume with a 50% ethanol-water solution to obtain reference stock solutions at the target concentrations. These solutions were stored at 4 °C for subsequent use.

#### 2.4.2. Preparation of Finished Infusions

On a clean bench, 20 mL of Tanreqing Injection was accurately measured and 1 g of meropenem for injection was accurately weighed. These were separately prepared using 100 mL and 250 mL of 0.9% sodium chloride injection (NS) as the solvent, respectively. The prepared infusions were stored at room temperature in a sealed container protected from light.

#### 2.4.3. Preparation of Test Solutions

Based on the therapeutic regimens recommended in drug package inserts and clinical guidelines, test solutions were prepared in the Pharmacy Intravenous Admixture Service (PIVAS) according to the schemes outlined in Table 1. Six experimental groups were established. For Groups A, B, D, and E, the finished infusions were evaluated immediately after preparation; 10 mL aliquots were collected at 0, 2, 4, 8, 12, and 24 h to monitor changes in appearance, pH, osmolality, and insoluble particle counts. For Groups C and F, 20 mL of Tanreqing injection was accurately measured and mixed with meropenem. These mixtures were compared with the negative control solution at 0 h, and 10 mL aliquots were collected at 0, 2, 4, 8, 12, and 24 h to determine changes in the aforementioned parameters.

To determine the contents of baicalin and meropenem, an appropriate volume of the mixed finished infusion was sampled, diluted 20-fold with the corresponding solvent, and filtered through a 0.22 μm membrane filter to obtain the test solution. For the determination of chlorogenic acid content, another portion of the mixed finished infusion was directly filtered through a 0.22 μm membrane filter without prior dilution. Three parallel replicates were prepared for each experimental group.

#### 2.4.4. Preparation of Negative Control Solution

A 20 mL aliquot of Tanreqing Injection was accurately measured and dissolved in 100 mL and 250 mL of 0.9% sodium chloride injection, respectively, to serve as negative controls.

### 2.5. Method Validation

#### 2.5.1. System Suitability

**Figure 1:**
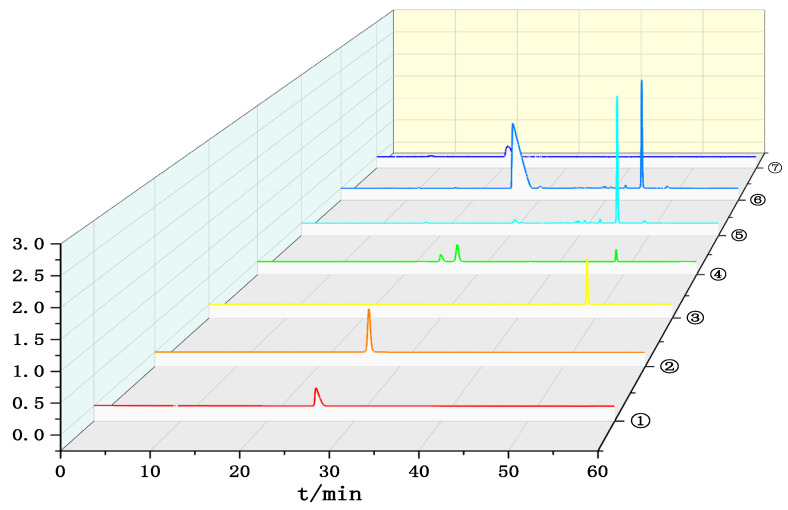
UPLC chromatogram of four substances. ① Meropenem reference standard; ② Chlorogenic acid reference standard; ③ Baicalin reference standard; ④ Mixed reference standard; ⑤ Mixed test solution; ⑥ Mixed test solution of Tanreqing Injection and meropenem; ⑦ Meropenem impurity A reference standard.

The chromatographic profiles of the reference standards and test solutions are shown in Figure 1. Appropriate volumes of the reference stock solution, mixed reference stock solution, and test solution were accurately measured and analyzed under the chromatographic conditions specified in Section 2.3.1. Chromatographic peaks for meropenem, chlorogenic acid, and baicalin were well-shaped. No interference was observed at the corresponding retention times, indicating high method specificity.

#### 2.5.2. Linearity

A series of standard working solutions were prepared from the mixed reference stock solution at the following concentrations: meropenem (1.0, 0.75, 0.5, 0.4, 0.3, 0.2, and 0.1 mg·mL^−1^), chlorogenic acid (0.75, 0.5, 0.4, 0.3, 0.2, 0.1, and 0.1 mg·mL^−1^), and baicalin (0.75, 0.5, 0.4, 0.3, 0.2, 0.1, and 0.05 mg·mL^−1^). These solutions were analyzed under the conditions specified in Section 3.1. Linear regression was performed by plotting the peak area (*Y*) against the mass concentration (*X*). The regression equations were as follows:

Meropenem: *Y* = 1.3 × 10^4^*X* − 1.5 × 10^5^ (*r* = 0.999)

Chlorogenic acid: *Y* = 3.6 × 10^4^*X* − 3.9 × 10^5^ (*r* = 0.998)

Baicalin: *Y* = 2.3 × 10^4^*X* − 1.1 × 10^6^ (*r* = 0.993)

Meropenem impurity A: *Y* = 1.6 × 10^3^*X* − 4.3 × 10^1^ (*r* = 0.999)

The results indicated excellent linearity for all three analytes within their respective concentration ranges.

The limits of detection (LOD) and limits of quantification (LOQ) for the analytes were established based on signal-to-noise (*S/N*) ratios of 3:1 and 10:1, respectively. The LODs for meropenem, chlorogenic acid, baicalin, and meropenem impurity A were determined to be 0.015, 0.012, 0.018, and 0.020 mg·mL^−1^, whereas their corresponding LOQs were 0.045, 0.036, 0.054, and 0.060 mg·mL^−1^.

To further verify the reliability of the method at the lower quantitative boundaries, precision at the LOQ levels was evaluated through six replicate injections. The relative standard deviations (RSDs) of the peak areas were 3.26%, 2.83%, 1.15%, and 3.79% for meropenem, chlorogenic acid, baicalin, and meropenem impurity A, respectively. All RSD values were well below 5.0%, confirming that the proposed analytical method possesses adequate sensitivity and maintains excellent instrumental precision even at trace concentration levels.

#### 2.5.3. Precision

The mixed reference stock solution was analyzed via six replicate injections under the conditions specified in Section 2.4.1. The RSD for the peak areas of meropenem, chlorogenic acid, and baicalin were 0.28%, 0.72%, and 0.97%, respectively (*n* = 6), indicating high instrumental precision.

#### 2.5.4. Repeatability

Six independent replicates of the test solutions from Groups B and D (Section 2.4.3) were prepared and analyzed. The peak area of each drug was recorded, and the RSD was calculated accordingly. The RSD values for the contents of meropenem, chlorogenic acid, and baicalin were 0.90%, 0.43%, and 0.21%, respectively (n = 6), demonstrating that the method is highly repeatable.

#### 2.5.5. Sample Recovery

Test solutions from Groups A and B (Section 2.4.3) were prepared in triplicate. Aliquots of 5 mL were transferred into 10 mL volumetric flasks, to which control standard solutions were added at levels corresponding to 80%, 100%, and 120% of those in the test solutions. The mixtures were diluted to volume, injected for determination, and the peak areas were recorded. Measured values were substituted into the standard curves to calculate sample recovery using the following formula:

$$Sample\;Recovery\;\left(\%\right)=\frac{C_{total}-C_{original}}{C_{added}}\times 100\%$$

where *C_total_* is the total measured content after standard addition, *C_original_* is the initial drug content before standard addition, and *C_added_* represents the added amount of the reference standards.

The results are summarized in Table 2. The average sample recoveries for the three components ranged from 99.14% to 103.25%, with all RSD values below 5.0% (n = 9), confirming the accuracy of the method.

### 2.6. Statistical Analysis

Statistical analysis was performed using SPSS 21.0. Data are presented as mean ± standard deviation. Comparisons between groups were conducted using independent samples *t*-tests, with *p* < 0.05 considered statistically significant.

## 3. Results

### 3.1. Appearance

**Figure 2:**
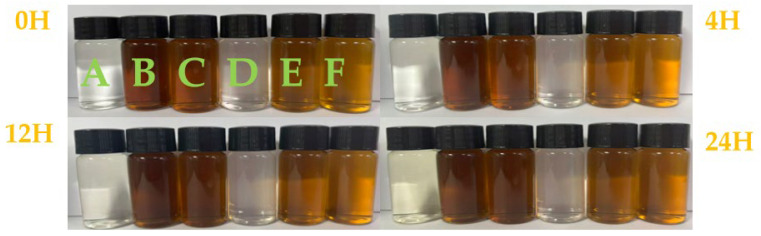
Appearance of experimental solutions at 0, 4, 12, and 24 h. Groups: (A–C) 100 mL NS; (D–F) 250 mL NS. (A,D) Meropenem alone; (B,E) Tanreqing alone; (C,F) Meropenem + Tanreqing. Note: All vials were colorless transparent glass and filled to the brim during visual assessment. The yellowish-brown coloration is intrinsic to Tanreqing Injection and varies in intensity according to dilution ratio (100 mL vs. 250 mL NS). No turbidity, precipitation, or abnormal physical changes were observed in any group.

In accordance with the Pharmacopoeia of the People’s Republic of China (2025 Edition, General Chapter 0901, 0902, and 0904) [22], all experimental solutions remained clear and free of turbidity, precipitation, or stratification throughout the observation period (0, 4, 12, and 24 h) at room temperature. No visible particulates were detected under a light intensity of 1500 lx at any time point (Figure 2).

### 3.2. pH and Osmolality

The pH and osmolality of the infusions were monitored over 24 h. The pH values across all groups ranged from 7.59 to 8.08, slightly exceeding the physiological range of human plasma (7.35–7.45). Osmolality ranged from 276 to 480 mOsmol·kg^−1^. According to the Expert Consensus on Physical Stability and Compatibility Inspection Indicators of Finished Infusions and relevant literature standards, the pH variation remained < 1.0 unit and the osmolality variation was <10% within 24 h for all six groups, indicating no incompatibility regarding these parameters [23]. The detailed pH and osmolality values for each experimental group over 24 h are shown in Table 3.

### 3.3. Insoluble Particles

**Figure 3:**
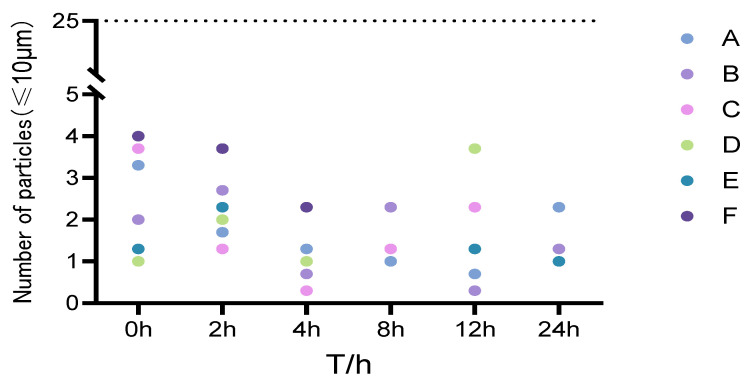
Distribution of insoluble particles ≥ 10 μm in mixed solutions. The dashed line in the figure represents the pharmacopoeial upper limit for insoluble particles (25 μm). No particles ≥ 25 μm were detected in any mixed solution within 8 h at room temperature.

Insoluble particles were evaluated according to the Pharmacopoeia of the People’s Republic of China (2025 Edition, General Chapter 0903) [22]. For intravenous injections ≥ 100 mL, the limits are ≤25 particles/mL for particles ≥ 10 μm and ≤3 particles/mL for particles ≥ 25 μm. After allowing samples to stand to remove air bubbles, five parallel measurements were performed; the first was discarded, and the average of the subsequent four was recorded. As shown in Figure 2, no particles ≥ 25 μm were detected in any mixed solution within 8 h at room temperature. The distribution of particles ≥ 10 μm is illustrated in Figure 3.

Number of insoluble particles (≥10 μm) in each experimental group over 24 h. The abscissa represents the observation time (h), and the ordinate represents the number of insoluble particles (mL^−1^).

### 3.4. Chemical Stability and Impurity Formation

Test solutions were filtered through a 0.22 µm membrane prior to injection. Peak areas were recorded and substituted into standard curves to calculate drug content. Impurity A, a characteristic degradation product of meropenem, was monitored as a core stability indicator. According to the Pharmacopoeia of the People’s Republic of China (2025 Edition) [22], the content of Impurity A must not exceed 1.0%.

Initial content was defined as follows: for Groups A, B, D, and E, the content at 0 h was set as the 100% baseline. For Groups C and F, the content of the negative control group at 0 h served as the 100% baseline for calculating relative percentages at subsequent time points.

Meropenem monotherapy groups (Groups A and D): In Group A (100 mL NS), the meropenem content decreased to 88.9% at 24 h (11.1% reduction), while Impurity A increased from 0.7% to 3.7% over time. Group D (250 mL NS) exhibited significantly superior stability; at 24 h, meropenem content remained at 94.2% (5.8% reduction), and Impurity A increased from 0.7% at 0 h to 2.6% at 24 h. These results confirm that higher dilution volumes improve meropenem stability.

Tanreqing monotherapy groups (Groups B and E): In Group B (100 mL NS), chlorogenic acid and baicalin contents decreased to 80.0% and 92.0% at 24 h, respectively. In Group E (250 mL NS), the degradation was less pronounced, with chlorogenic acid at 85.2% and baicalin at 94.9% at 24 h. This indicates that 250 mL NS better maintains the stability of Tanreqing’s active ingredients.

Meropenem–Tanreqing combination groups (Groups C and F): Significant degradation of chlorogenic acid occurred immediately upon mixing (0 h), particularly in the 100 mL NS group. In Group C, chlorogenic acid content dropped to 95.7% at 0 h and further to 28.1% at 24 h (71.9% total reduction). In Group F, it decreased to 97.5% at 0 h and 56.0% at 24 h (44.0% total reduction). Meropenem also exhibited more significant, time-dependent degradation in combination than in monotherapy. In Group C, meropenem content decreased to 65.2% at 24 h, while in Group F, it dropped to 85.8% (14.2% reduction). Furthermore, Impurity A levels far exceeded the 1.0% limit in both combination groups, with a more rapid increase in the 100 mL NS group. In Group C, Impurity A was 1.0% at 0 h and reached 13.3% ± 1.4% at 24 h (13.3 times the limit). In Group F, Impurity A was 1.1% at 0 h, rose to 6.0% at 8 h, and reached 10.8% at 24 h (10.8 times the limit). These results indicate that the lower-concentration infusion was relatively more stable than its high-concentration counterpart.The relative content changes of Meropenem, Impurity A, Chlorogenic Acid, and Baicalin in each group are shown in Table 4.

## 4. Discussion

### 4.1. Rationale for Dose and Solvent Selection

The package inserts for both meropenem for injection and Tanreqing Injection recommend 5% glucose injection or NS as suitable vehicles. However, the relatively low pH of 5% glucose injection (typically 3.2–5.5) has been reported to induce turbidity when mixed with Tanreqing Injection [24]. Consequently, NS is more frequently used as the diluent in clinical practice [25]. To ensure clinical relevance, this study investigated the compatibility of 1 g of meropenem and 20 mL of Tanreqing Injection, each diluted in either 100 mL or 250 mL of NS, representing standard therapeutic regimens for SP. These findings provide direct evidence-based guidance for the preparation and administration of these intravenous medications.

### 4.2. Optimization and Validation of Chromatographic Conditions

During the optimization of the HPLC method, various mobile phase systems with differing pH values were evaluated. An acetonitrile—0.01 mol/L potassium dihydrogen phosphate system (adjusted to pH 2.5 with phosphoric acid) was ultimately selected. This system enabled the simultaneous determination of meropenem, chlorogenic acid, and baicalin at a wavelength of 318 nm, providing excellent separation and high specificity. Method validation—including system suitability, linearity, precision, repeatability, and recovery—confirmed that the analytical procedure met the stringent requirements for stability studies, thereby ensuring the reliability of the quantitative results.

### 4.3. Mechanistic Insights into the Incompatibility Between Meropenem and Tanreqing Injection

This study represents the first systematic characterization of the in vitro incompatibility between meropenem for injection and Tanreqing Injection. The rapid degradation of chlorogenic acid and the sharp increase in meropenem Impurity A upon mixing suggest a potent chemical interaction. Given the complex phytochemical profile of TCM injections, the Guiding Principles for Clinical Application of Traditional Chinese Medicine Injections explicitly stipulate that TCM injections be administered independently and used in combination with other drugs only with extreme caution. Our results provide a rigorous physicochemical basis for adhering to these safety principles.

The molecular mechanism underlying this interaction is hypothesized to involve the β-lactam ring of meropenem, which serves as a highly reactive electrophilic center. Under specific pH conditions, this ring may undergo opening or acylation reactions with the phenolic acid moieties of chlorogenic acid. The alkaline nature of the Meropenem solution (pH ~8.0) likely facilitates the deprotonation of chlorogenic acid’s phenolic hydroxyl groups, making it more susceptible to oxidative degradation or nucleophilic attack on the Meropenem β-lactam ring. This finding underscores the critical need for compatibility studies between carbapenem antibiotics and TCM injections. Future research utilizing structural identification techniques, such as mass spectrometry, is required to definitively elucidate these reaction products [26].

### 4.4. Clinical Significance and Practical Implications

SP progresses rapidly and frequently leads to life-threatening complications, including bloodstream infections, multiple organ dysfunction syndrome (MODS), and septic shock. Meropenem remains a cornerstone of broad-spectrum anti-infective therapy due to its potent activity against pathogens such as Klebsiella pneumoniae, Escherichia coli, and Pseudomonas aeruginosa [27]. In the context of rising meropenem resistance, the adjunctive use of TCM can provide synergistic effects in anti-infection, heat-clearing and detoxifying, dispersing lung qi and resolving phlegm, while potentially reducing the adverse effects of meropenem and improving clinical efficacy. Baicalin and chlorogenic acid, the active components in Tanreqing Injection, possess broad-spectrum antimicrobial, anti-inflammation, antipyretic and immune regulation. Their combination with antibiotics can enhance biological activity and antibiotic sensitivity while potentially reducing drug toxicity [28,29,30,31]. Clinically, the combined use of meropenem and Tanreqing Injection aims to alleviate inflammatory injury, accelerate symptom relief, and improve respiratory function [32,33].

Although the compatibility results of this study do not support the parallel mixed infusion of these two agents, the findings address a critical research gap regarding the interaction between antibiotics and TCM injections. These results provide theoretical guidance for optimizing clinical administration. To ensure patient safety and therapeutic efficacy, clinicians must account for this incompatibility. Furthermore, this study provides clinical pharmacists with the necessary data to guide rational drug use in hospital settings.

### 4.5. Study Limitations and Future Perspectives

This study provides physicochemical evidence of the incompatibility between meropenem and Tanreqing injection, but several limitations should be noted. The static in vitro design does not capture the conditions typically encountered in the ICU. Patients with SP often present with fluctuating hemodynamics and body temperature, and frequently receive multiple intravenous therapies simultaneously through Y-site connectors or multi-lumen catheters. These dynamic flow and mixing conditions were not represented in our study. Additionally, although the use of generic meropenem from the national volume-based procurement program reflects current clinical use, potential differences in excipients compared with the originator product were not examined. Environmental factors such as prolonged light exposure and high temperature were not evaluated and may influence degradation behavior to some extent. In conclusion, the present findings provide a preliminary physicochemical basis for understanding incompatibility but do not capture the complexity of in vivo conditions. Future studies should incorporate dynamic compatibility assessments using simulated Y-site models. In addition, well-designed randomized controlled trials and in vivo pharmacokinetic evaluations are needed to better inform safe administration practices in critically ill patients.

By simulating clinically relevant doses and infusion conditions, this study systematically investigated the stability and compatibility of meropenem and Tanreqing Injection in NS over 24 h. The findings clarify the chemical incompatibility between these two agents and provide essential data to support safe medication practices for SP.

Meropenem monotherapy exhibited good stability in NS; when 1 g was dissolved in 100 mL or 250 mL of NS, the content remained at 88.9% (*v*/*v*) and 94.2% (*v*/*v*), respectively, after 24 h. In contrast, Tanreqing Injection monotherapy showed lower stability in NS. When 20 mL was dissolved in 100 mL of NS, chlorogenic acid content decreased to 80.0% (*v*/*v*) at 24 h, whereas in 250 mL of NS, it remained at 85.2% (*v*/*v*). Compatibility significantly decreased upon mixing: chlorogenic acid levels began to decline immediately and dropped to 28.1% (*v*/*v*) (100 mL NS) and 56.0% (*v*/*v*) (250 mL NS) after 24 h. Simultaneously, meropenem Impurity A increased significantly, with more pronounced growth in the 100 mL NS group (*p* < 0.05).

Clinically, to maintain the structural integrity of the active constituents, a 250 mL NS diluent is strongly preferred for both agents. If a 100 mL dilution is utilized, the solutions must be administered promptly and the infusion duration strictly minimized to prevent sub-threshold degradation. Most importantly, the simultaneous parallel infusion of meropenem and TRQ is strictly contra-indicated due to the rapid accumulation of toxic impurities. In ICU settings where limited venous access necessitates sequential administration via a shared line, it is imperative that the intravenous tubing and Y-site connectors be rigorously flushed with a compatible neutral vehicle (e.g., NS) between infusions. Adherence to these evidence-based administration protocols is essential to mitigate the risks of undocumented chemical interactions and to ensure maximum patient safety in critical care environments.

## Figures and Tables

**Table 1:** Experimental Grouping and Composition of Clinical Infusion Regimens for Tanreqing Injection and Meropenem.

Solvent/Solvent Volume	Group	Component
100 mL NS	A	1 g Meropenem
100 mL NS	B	20 mL Tanreqing Injection
100 mL NS	C	20 mL Tanreqing Injection + 1 g Meropenem
250 mL NS	D	1 g Meropenem
250 mL NS	E	20 mL Tanreqing Injection
250 mL NS	F	20 mL Tanreqing Injection + 1 g Meropenem

Abbreviations: NS, 0.9% sodium chloride injection.

**Table 2:** Recovery Results for Meropenem, Chlorogenic Acid, and Baicalin.

	Spiked Concentration Level/(μg·mL^−1^)	Spiked Recovery	Average Recovery	RSD	Average RSD
Meropenem	120%	99.81	99.63%	0.35	0.74%
	100%	99.31		0.87	
	80%	99.78		1.01	
Chlorogenic Acid	120%	100.2%	99.65%	1.42%	1.79%
	100%	99.61%		2.71%	
	80%	99.14%		1.24%	
Baicalin	120%	99.20%	101.23	0.90%	0.96%
	100%	101.23%		0.97%	
	80%	103.25%		1.00%	

Abbreviations: RSD, relative standard deviation.

**Table 3:** pH and osmolality of experimental groups over 24 h.

Experimental Group	pH Value (Mean ± Sd)					
	0 h	2 h	4 h	8 h	12 h	24 h
A	8.03 ± 0.02	8.03 ± 0.01	7.95 ± 0.01	7.92 ± 0.01	7.91 ± 0.03	7.89 ± 0.01
B	7.85 ± 0.01	7.85 ± 0.02	7.67 ± 0.01	7.62 ± 0.00	7.61 ± 0.01	7.58 ± 0.01
C	7.90 ± 0.03	7.80 ± 0.01	7.80 ± 0.01	7.80 ± 0.01	7.82 ± 0.00	7.78 ± 0.01
D	8.10 ± 0.01	8.02 ± 0.03	8.00 ± 0.01	7.93 ± 0.01	7.90 ± 0.01	7.89 ± 0.02
E	7.79 ± 0.01	7.71 ± 0.01	7.62 ± 0.01	7.59 ± 0.01	7.56 ± 0.00	7.57 ± 0.01
F	8.00 ± 0.01	7.95 ± 0.01	7.94 ± 0.01	7.81 ± 0.01	7.82 ± 0.01	7.79 ± 0.02
Experimental Group	Osmolality/(mOsmol·kg^−1^) (mean ± sd)					
	0 h	2 h	4 h	8 h	12 h	24 h
A	314.3 ± 0.01	309.2 ± 0.00	315.4 ± 0.02	319.0 ± 0.01	315.5 ± 0.01	315.7 ± 0.03
B	428.2 ± 0.00	429.8 ± 0.03	422.0 ± 0.01	423.0 ± 0.00	423.3 ± 0.02	423.6 ± 0.01
C	473.4 ± 0.01	479.0 ± 0.01	471.1 ± 0.01	478.0 ± 0.01	478.1 ± 0.01	478.2 ± 0.02
D	280.3 ± 0.02	276.9 ± 0.01	286.0 ± 0.00	282.0 ± 0.00	283.7 ± 0.03	274.2 ± 0.01
E	332.2 ± 0.01	331.0 ± 0.02	335.4 ± 0.01	333.1 ± 0.01	330.2 ± 0.01	328.4 ± 0.03
F	347.7 ± 0.01	354.3 ± 0.01	349.0 ± 0.01	353.7 ± 0.01	352.7 ± 0.01	354.1 ± 0.01

**Table 4:** Relative Content of Meropenem, Impurity A, Chlorogenic Acid, and Baicalin in Each Experimental Group.

Group	Drug	Relative Percentage Content of Drug (%, Mean ± Sd)						
		Negative Control Group	0 h	2 h	4 h	8 h	12 h	24 h
A	Meropenem	——	100	99.7 ± 0.3	98.2 ± 1.4	97.9 ± 0.0	92.2 ± 1.8	88.9 ± 1.8
	Impurity A	——	0.7 ± 8.2	1.2 ± 4.1	1.6 ± 5.0	2.1 ± 4.2	2.6 ± 3.0	3.7 ± 2.0
B	Chlorogenic Acid	——	100	97.2 ± 3.1	91.6 ± 3.9	86.8 ± 3.8	84.0 ± 3.6	80.0 ± 3.0
	Baicalin	——	100	97.4 ± 0.1	96.0 ± 0.3	94.3 ± 0.3	94.2 ± 0.2	92.0 ± 0.3
C	Meropenem	——	100	96.5 ± 0.7	93.9 ± 0.3	87.1 ± 0.1	79.0 ± 0.1	65.2 ± 0.3
	Impurity A	——	1.0 ± 0.1	3.0 ± 0.7	4.7 ± 1.9	7.2 ± 0.3	9.0 ± 1.4	13.3 ± 1.4
	Chlorogenic Acid	100	95.7 ± 0.8	81.8 ± 0.6	73.9 ± 0.8	53.6 ± 0.6	36.0 ± 2.0	28.1 ± 0.7
	Baicalin	100	99.6 ± 0.6	96.8 ± 0.4	96.7 ± 0.1	95.2 ± 0.5	92.7 ± 1.0	90.1 ± 1.5
D	Meropenem	——	100	99.8 ± 0.2	97.4 ± 1.4	96.5 ± 1.5	94.8 ± 0.4	94.2 ± 0.7
	Impurity A	——	0.7 ± 1.4	0.9 ± 0.8	1.2 ± 2.2	1.8 ± 0.5	2.2 ± 4.0	2.6 ± 3.4
E	Chlorogenic Acid	——	100	97.2 ± 0.1	93.5 ± 0.3	90.2 ± 0.5	86.8 ± 0.3	85.2 ± 0.3
	Baicalin	——	100	99.6 ± 0.6	98.4 ± 0.4	97.0 ± 0.5	95.8 ± 0.5	94.9 ± 0.4
F	Meropenem	——	100	96.1 ± 0.2	94.6 ± 0.2	90.5 ± 0.2	87.4 ± 0.2	85.8 ± 0.8
	Impurity A	——	1.1 ± 4.1	3.0 ± 3.5	4.3 ± 4.6	6.3 ± 3.8	7.4 ± 1.8	10.8 ± 1.8
	Chlorogenic Acid	100	97.5 ± 0.8	85.9 ± 0.2	78.5 ± 0.3	69.8 ± 0.2	64.0 ± 0.8	56.0 ± 0.6
	Baicalin	100	99.3 ± 0.2	97.3 ± 0.2	95.9 ± 0.4	92.3 ± 0.5	91.4 ± 0.5	89.1 ± 0.3

Note: According to the Pharmacopoeia of the People’s Republic of China (2025 Edition) [22], the limit for meropenem Impurity A is ≤1%; “—” indicates no corresponding data in the negative control group.

## Data Availability

The original contributions presented in this study are included in the article. Further inquiries can be directed to the corresponding author.
